# Case Report: Renal sarcoidosis coexisting with membranous and IgA nephropathy, a very uncommon association

**DOI:** 10.3389/fneph.2026.1809424

**Published:** 2026-07-13

**Authors:** Mariel Hernández-Pérez, Daniel Enos, Rocío Contreras Faundez, Joaquín Ramírez Herrera, Pablo Tapia Riedemann, Gonzalo P. Méndez, Jorge Gutiérrez Ventura

**Affiliations:** 1 Internal Medicine Department, Universidad de Concepción, Los Ángeles, Biobio, Chile; 2 Nephrology Department, Hospital Víctor Ríos Ruiz, Los Ángeles, Biobio, Chile; 3 Pathology Unit, Laboratorio Inmunocel, Santiago, Chile

**Keywords:** IgA nephropathy, impure nephrotic syndrome, interstitial nephritis, membranous nephropathy, renal sarcoidosis, steroids response

## Abstract

**Introduction:**

Sarcoidosis is a multisystemic inflammatory disease of unknown etiology with more frequent lung and lymph node involvement. Despite kidney involvement occurring between 25% and 43%, it may be underdiagnosed, with interstitial granulomatous nephritis and nephrocalcinosis being the most common features, and glomerular disease being less frequent.

**Case report:**

A 43-year-old man came to the emergency room (ER) because of macroscopic hematuria, arthralgias, pitting inferior extremities edema, asthenia, and dry cough without dyspnea. Evaluation revealed nephrotic range proteinuria and glomerular hematuria, suggesting an impure nephrotic syndrome. The kidney biopsy showed features of membranous nephropathy and granulomatous interstitial nephritis, with immunofluorescence showing codominant mesangial immunoglobulin A (IgA) and immunoglobulin G (IgG) deposits.

**Management and outcome:**

Steroids plus angiotensin receptor blocker (ARB) antagonists were effective, with symptoms subsiding and proteinuria level decreasing after 8 months of follow-up.

## Introduction

Sarcoidosis is a multisystemic inflammatory disease of unknown etiology (1, 2), affecting predominantly middle-aged people (1) of either gender. It is believed that it has a genetics base and is triggered after environmental exposure to potentially toxic elements such as inorganic dust (3). Histologically, it appears as a non-caseating granuloma surrounded by giant multinucleated epithelioid cells (2). Even though many different organs could be involved, in more than 90% of the cases, lung and mediastinal lymph nodes are the most affected (1). Additionally, renal involvement frequency oscillates between 25% and 43%, and is often misdiagnosed (2), owing to a variety of associated renal sarcoidosis diseases such as interstitial granulomatous nephritis, the most frequent, and also secondary hypercalcemic nephrocalcinosis (2). Although renal involvement is often missed, kidney biopsy remains the diagnostic gold standard. Furthermore, sarcoidosis glomerular disease can include secondary membranous nephropathy (4–7), minimal change disease (8–10), focal and segmentary glomerular sclerosis (11, 12), and immunoglobulin A (IgA) nephropathy (13–15). Herein, we describe a case report witnessing the coexistence of two different glomerular diseases associated with the diagnosis of sarcoidosis, predominating the nephrotic one.

## Case report

A 43-year-old Hispanic man with chronic occupational exposure to silica presented with macrohematuria and foamy urine, mild arthralgias, leg swelling with few painful red skin nodules located in both pretibial zones, clinically diagnosed as erythema nodosum, asthenia, and non-productive cough. He denied a history of fever, weight loss, night sweats, hemoptysis, or dyspnea. Evaluated by a nephrologist, he was found to be hypertensive, with erythema nodosum and pitting edema in both legs, with scarce bilateral lung rales, more prominent on the right base, without palpable lymph nodes. Laboratory findings highlighted the following: serum creatinine, 0.86 mg/dL; calcium, 9.5 mg/dL; low-density cholesterol, 168 mg/dL; albumin, 2.8 g/dL; 24-h urinary protein, 3.5 g; hemoglobin, 16.5 g/dL; and over 100 isomorphic red cells per high-power field without other relevant findings in the urine sample. Subsequently, an immunologic panel was carried out, showing antinuclear antibodies (ANA)-positive 1/80 fine granular nuclear pattern and anti-Ro 192.6 U/mL [normal value (NV) of less than 30 U/mL], without sicca symptoms, whereas other autoantibodies such as extractable nuclear antibodies (ENA), P and C antineutrophil cytoplasmic antibodies (P-ANCA and C-ANCA), myeloperoxidase (MPO), proteinase 3 (PR3), B and C hepatitis, VDRL, anti-phospholipase A2 receptor (PLA2R1), Tuberculosis Mycobacterium QuantiFERON, anti-streptolysin O, and HIV test were all negative, without the possibility to request serum angiotensin-converting enzyme. A thoracic computed tomography (CT) scan revealed many bilateral small lung nodules of less than 1 cm, especially in the upper lobules, mild expiratory air trapping, and many lymph nodes located in paratracheal, below carina, sub-aortic, para-aortic, and both pulmonary hila, ruling out active infection due to the absence of symptoms, fever, and normal inflammatory parameters, such as C-reactive protein and procalcitonin.

**Figure 1:**
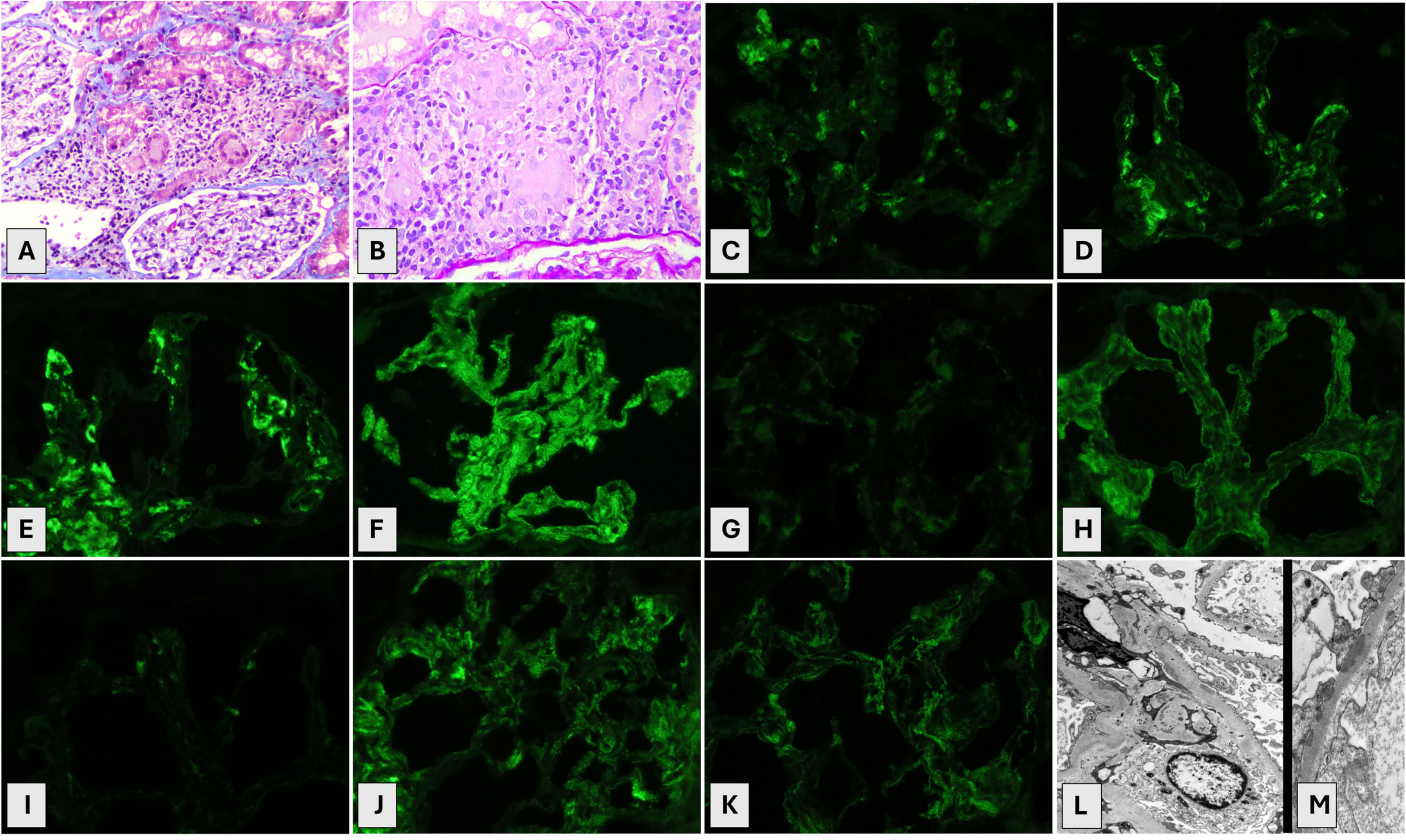
**(A)** Kidney cortex shows a prominent granuloma composed of multinucleated giant cells, histiocytes, and lymphocytes partially surrounding both glomeruli. The glomeruli reveal hypertrophy, but their architecture is preserved (Masson Trichrome stain, 200×). **(B)** Sarcoid-type granuloma composed of multinucleated giant cells intermingled with numerous lymphocytes with a peripheral arrangement. Emperipolesis is also recognized (PAS stain, 400×). **(C–E)** C3, C1q, and IgA, respectively, showing deposits mostly in the mesangial zones. **(F)** IgG stain showing fine granular deposits in capillary loops in a segmental pattern. **(G)** Anti-PLA2R1 is negative. **(H–K)** IgG subclasses (1, 2, 3, and 4), with fine granular deposits of IgG1, IgG3, and IgG4 capillary loops with a segmental pattern. IgG2 is negative (immunofluorescence microscopy, 400×). **(L, M)** Electron microscopy. **(L)** Segment of capillary tuft showing small electron-dense deposits in the mesangium (black arrows) and subepithelial zones (red arrows). **(M)** Glomerular basement membrane with several small, subepithelial electron-dense deposits, consistent with stage I of a membranous lesion (uranyl-acetate, lead-citrate; L: 6,600×; M: 9,000×).

Kidney biopsy was performed 4 weeks after. Three different tissue fragments were obtained and processed for light, immunofluorescence, and electron microscopy analysis. A total amount of 24 glomeruli was present considering the full sample, with two of them globally sclerotic and obsolete (8%). The light microscopy tissue sample was studied with hematoxylin–eosin, periodic acid–Schiff (PAS), Masson Trichrome, and silver-methenamine stains. It contained 15 glomeruli, 2 of which were obsolete. The remaining glomeruli had a mostly preserved architecture, revealing only mild to moderate thickening of capillary loops without spike formation, as confirmed by the histochemical stains (silver, Masson, and PAS). There was no evidence of hypercellular lesions; specifically, cellular crescents, endocapillary proliferation, or mesangial proliferative changes were recognized. Other lesions like segmental sclerosis, necrosis, wire loops, or thrombi were also absent. The tubulo-interstitial compartment had no significant tubular atrophy or fibrosis (less than 10%). It showed multifocal inflammatory infiltrates with lymphocytes, scattered eosinophiles, and several rounded, well-formed, and non-caseating epithelioid granulomas with multinucleated giant cells. Through immunofluorescence, frozen tissue sections were studied with a panel of 13 antibodies [C3, C1q, IgA, IgG, IgM, fibrinogen, kappa and lambda light chains, PLA2R1, and IgG subclasses (gamma heavy chains 1, 2, 3, and 4)]. The sample contained six glomeruli. IgG revealed fine granular deposits along peripheral capillary loops in a segmental but multifocal pattern of distribution, with polytypic expression of both light chains. IgG subclasses 1, 3, and 4 were also positive in the form of fine granules along capillary segments. C3, C1q, and PLA2R1 were negative for this distribution. The analysis also revealed IgA-positive, fine granular deposits at mesangial zones exclusively, with polytypic expression of both light chains and focal, lower-intensity C3, IgM, and C1q mesangial reactivities. The electron microscopy analysis was performed in one glomerulus, which revealed small to medium-sized, multiple subepithelial electron-dense deposits at capillary loops, in a segmental membranous pattern of distribution and without significant spike formation. Podocyte foot processes were effaced in more than 70% of the glomerular surface, consistent with secondary, intense, cytoplasmic injury of the visceral epithelial cells. No subendothelial deposits were recognized. The mesangium zones contained small electron-dense deposits, without an increase in matrix or cellularity. Tubulo-reticular inclusions were not seen (**Figure 1**).

## Treatment and outcome

We decided to start 30 mg of daily prednisone (0.5 mg/kg/day); meanwhile, the biopsy report was available, because active sarcoidosis was our first diagnostic approach, along with an angiotensin receptor blocker. After the biopsy report, we continued with our therapy scheme because, despite the IgA deposits, they seemed to be only an epiphenomenon, with no pathological significance in our case. Meanwhile, the patient was showing excellent response and was followed in our outpatient office. After a 3-month-period, we began tapering the prednisone dose, and after 13 months of follow-up, he was in 5 mg of oral steroids, having disappeared cough, erythema nodosum, and macrohematuria, whereas serum albumin reached normal levels. However, as regards proteinuria, it took 12 months to achieve an albumin-to-creatinine ratio (ACR) of less than 500 mg/g, with the patient maintaining permanently normal renal function (**Table 1**). Moreover, in the thoracic CT scan, the number of pulmonary nodules was notoriously diminished, showing less air trapped and smaller lymph nodes. Nowadays, he continues receiving the same treatment and is being monitored in the nephrology outpatient clinic.

**Table 1:** Laboratory findings at diagnosis and follow-up.

Laboratory/date	Baseline	Follow-up		
		3 months	8 months	12 months
Hemoglobin (g/dL)	16.5	15.3	17.2	15.7
Creatinine (mg/dL)	0.86	0.81	0.82	0.8
Serum albumin (g/dL)	2.8	4.0	NR	4.27
24-h Proteinuria (g/day)	3.5	NR	NR	0.682
ACR (mg/g)	NR	1,094	1,107	271
Urine red cells (per field)	More than 100	5–10 per field	0–2	0–2

ACR, albumin-to-creatinine ratio; NR no record.

## Discussion

The differential diagnosis in our case started with the clinical and radiologic presentation. Our first thought was minimal change disease associated with a lymphoproliferative disease, ruled out with renal biopsy findings and the diminished size of lymph nodes after an intermediate dose of prednisone. Other autoimmune diseases were suspected because of ANA and anti-Ro positivity, yet a Sjögren syndrome diagnosis was dismissed because of the absence of sicca symptoms, whereas all other autoimmune markers were negative. In addition, another important disease to consider was pulmonary tuberculosis (TBC), as it is a common infection in our country with a feasible secondary renal involvement, yet the negative QuantiFERON test, the absence of B symptoms and sputum, in addition to the nephrotic presentation of renal disease, ruled out that etiology. In fact, renal TBC commonly presents as a granulomatous interstitial nephritis with caseum as a central necrotic characteristic, with or without urinary tract obstructive granulomas, not seen in renal biopsy and renal CT scan, respectively. Completing the possible differential diagnoses, lung metastatic cancer had a very remote chance to explain the clinical and radiological picture, because of the absence of constitutional signs, the aspect of the pulmonary CT scan, the rapid resolution of lung nodules, and the decreasing lymph node size after prednisone therapy.

Upon checking the literature, the exact prevalence of renal sarcoidosis is unknown, with some reports varying from 0.3% to 3.5% (1, 2); therefore, clinical representations of renal involvement are heterogeneous and frequently diagnosed after pulmonary disease. This report presents a patient with silica exposure, which has increased risk for developing sarcoidosis, having an impure nephrotic syndrome, and finding an unusual association between renal sarcoidosis and its classic interstitial pattern with a secondary PLA2R-negative membranous glomerulonephritis. In addition, this association has been previously described by other authors as being caused by sarcoidosis (4–6), with a possible role of T4 and T8 lymphocytes from the granuloma in the glomerular damage pathogenesis (16, 17).

Moreover, the early renal involvement in sarcoidosis as a nephrotic syndrome was reported in a few published cases (18, 19), achieving diagnosis only by renal biopsy. This involvement is thought to appear many years after pulmonary diagnosis and management, as was described in 42% of 26 cases in one report, with renal sarcoidosis disease occurring at an average latency of 9.7 years following the first diagnosis (18). However, another author reported that 23% of sarcoidosis renal involvement was on average almost 8 years prior to sarcoidosis diagnosis, remaining as a hidden kidney disease, whereas only 35% of renal and lung lesions appeared simultaneously (19).

Thus, many authors affirm that all the immune processes responsible for sarcoidosis glomerular involvement are produced by sarcoidosis granulomas, owing to the increase of local cytokines in renal tissue, since the increased TNF-α levels produced by macrophages and activated T lymphocytes from sarcoidosis granulomas may provoke progressive injury and damage to the filtration barrier (16, 20). In this way, membranous deposits are the most frequent association between sarcoidosis and glomerular disease, reaching between 30% and 40% of cases (4–6). As an exception, Knehtl et al. described a case with sarcoidosis and positive phospholipase A2 receptor antibody (anti-PLA2R), suggesting that primary and secondary forms of the disease may share some physiopathologic process not yet clarified (20, 21).

Even though sarcoidosis and membranous nephropathy may be two independent entities in our case, the rapid response to an intermediate dose of oral steroids with full resolution of nephrotic syndrome strongly supports the causality relationship between both pathologies. Delving deeper, Ding et al. checked genetic features, finding that both diseases share common alleles of susceptibility (20), making it possible to develop both pathologies at the same time, maybe after being exposed to external noxa, which has not yet been well identified in our case.

In addition, Mahévas et al. reported that the first renal lesion in sarcoidosis was due to interstitial nephritis in 81% of 47 patients (19), remarking that glomerular involvement was with high probability produced by the immunologic activity of granuloma cells, as discussed previously (16, 17, 19).

Regarding the IgA mesangial deposits found in renal biopsy, it is thought that cytokines could play a crucial role over glycosylated systemic IgA overproduction, leading to the characteristic deposits of this glomerulopathy [22]. However, in our case, those deposits were not associated with remarkable inflammatory and proliferative mesangial lesions, making them unlikely to have an important participation in active disease, since their pathogenic role in this case was probably negligible. Although both pathologies may have a common molecular pathogenic mechanism, this association remains uncertain, because no specific antigen or antibodies shared by both diseases have been identified to date.

Analyzing the response to treatment, it was probably dependent on sarcoidosis interstitial nephritis control, given the excellent resolution of nephrotic syndrome with just a moderate dose of oral steroids. In addition, it is expected that therapy response will have an inverse relationship with the interstitial fibrosis extension found in the biopsy (18), which was mild in our patient, helping to achieve the almost immediate relief of nephrotic syndrome. The exception was the remaining elevated ACR for a few months, decreasing after 1 year. This topic will be better clarified with the patient being followed up, especially with the potential further relapses and new episodes of nephrotic proteinuria.

Finally, the clinical characteristics for sarcoidosis diagnosis in both renal involvement and the involvement of other organs must be remarked, to achieve an adequate diagnostic approach, keeping renal biopsy in mind as a major factor for histopathological confirmation and choosing the correct therapy.

## Conclusions

This paper depicts an impure nephrotic syndrome with concurrent pulmonary sarcoidosis,finding at renal biopsy the typical interstitial granulomas, with many IgG deposists in membranous location (stage I), as an unusual association, companied by mesangial IgA deposits with scarce inflammatory response, with probably less importance in pathogenesis cascade. These findings do not ensure a causal relationship, but it is interesting to consider in further investigations. The most important feature shown in our case was the high proteinuria and hypoalbuminemia returning rapidly to normal ranges after a short course of moderate dose of steroids, with the biopsy report confirming the sarcoidosis etiology, including the posterior disappearance of inflammatory red cells from urinary sediment and the renal profile almost achieving normal levels, and attenuating pulmonary CT scan findings.

## Limitations

We only reported one case with all the peculiar findings shown; thus, we cannot generalize our conclusions to other cases with such a small sample.

## Data Availability

The raw data supporting the conclusions of this article will be made available by the authors, without undue reservation.
